# Effects of a Macro-Nutrient Preload on Type 2 Diabetic Patients

**DOI:** 10.3389/fendo.2015.00139

**Published:** 2015-09-16

**Authors:** Chun-Jun Li, Gunnar Norstedt, Zhao-Gian Hu, Pei Yu, Dai-Qing Li, Jing Li, Qian Yu, Magnus Sederholm, De-Min Yu

**Affiliations:** ^1^Key Laboratory of Hormone and Development (Ministry of Health), Department of Endocrinology, 2011 Collaborative Innovation Center of Tianjin for Medical Epigenetics, Tianjin Institute of Endocrinology, Metabolic Disease Hospital, Tianjin Medical University, Tianjin, China; ^2^Center for Molecular Medicine, Karolinska Institutet, Stockholm, Sweden; ^3^Department of Women’s and Children’s Health, Karolinska Institutet, Stockholm, Sweden

**Keywords:** diabetes type II, nutrition, preload, human, glucose tolerance

## Abstract

**Aims:**

Macro-nutrient preloads given 30 min before regular meals may improve metabolism. The aim was to investigate how type 2 diabetic patients react to a preload consisting of a blend of macro-nutrients with a low-glycemic index (Inzone Preload^®^).

**Methods:**

In a before–after study design, 30 subjects with type 2 diabetes mellitus (T2DM) were enrolled in a 12-week program. All subjects were given Inzone Preload (43% proteins, 29% carbohydrates, 10% lipids, and 9% fibers, 71 kcal), 30 min before each meal during 12 weeks. Fasting glucose and postprandial 2 h glucose were monitored every second week. Body weight (BW) and waist circumference were measured each month. Fasting plasma glucose, glycosylated hemoglobin, serum lipids, fasting insulin, C-reactive protein, and homeostasis model assessment were evaluated before and after the intervention. Subjective appetite was monitored using visual analogue scales after the Inzone Preload.

**Results:**

The dietary intervention significantly influenced several metabolic parameters compared to base line. Inzone Preload treatment reduced mean postprandial plasma glucose levels (12.2 ± 1.2 vs. 10.5 ± 2.0 mmol/L), HbA1c (7.4 ± 0.3 vs. 7.1 ± 0.2%), mean total cholesterol (4.8 ± 0.9 vs. 4.3 ± 0.8 mmol/L), low-density lipoprotein cholesterol (2.8 ± 0.6 vs. 2.5 ± 0.4 mmol/L), and CRP (1.5 ± 1.4 vs. 0.7 ± 0.7 mg/L). BW loss of more than 3% was seen in 13 participants (43%). Feelings of satiety were significantly higher after Inzone Preload than after habitual breakfast (*p* < 0.05). No significant changes in fasting blood glucose, high-density lipoprotein and total triacylglycerol, HOMA-IR, and HOMA-β were observed.

**Conclusion:**

A macro-nutrient preload treatment reduces postprandial glucose, inflammatory markers, and serum lipids in patients with T2DM. Approximately half of the study group also displayed reduced BW.

## Introduction

Diabetes mellitus is a serious chronic disease threatening human health. It is estimated that 382 million people worldwide are affected and that the numbers will continue to increase to 471 million by 2035 (1). The prevalence of diabetes has increased significantly in recent decades and is now reaching epidemic proportions in China (2, 3). More recently, a study estimated that the prevalence of diabetes is 11.6% and pre-diabetes affects 50.1% of the adult Chinese population (4). Therefore, the need for further research on primary prevention and control of diabetes is of paramount importance.

The majority of patients with impaired glycemic control do not meet the recommended criteria for pharmacological treatment, exposing them to risks of sustained hyperglycemia (2). There is increasing evidence that postprandial hyperglycemia plays a major role in the pathogenesis of diabetic macro-vascular complications (5, 6). Therapeutic strategies to reduce postprandial glucose are therefore of fundamental importance in the management of T2DM (7, 8). For patients with mild-to-moderate hyperglycemia, postprandial blood glucose is a better predictor of HbAlc than fasting blood glucose (FBG). Accordingly, pharmacological and dietary strategies to decrease postprandial glycemia are receiving increased attention (9, 10). Major determinants of postprandial blood glucose concentrations are the rate of gastric emptying and the postprandial insulin response (6). Therefore, modulation of gastric emptying and enhancement of insulin effects to minimize dietary postprandial glucose elevation has a potential to optimize glycemic control in diabetes. Over the past two decades, diets based on foods that can reduce postprandial blood glucose excursions have been investigated. Several studies have demonstrated that low-glycemic index (GI) diets are useful in the protection of diabetes (11–13), and low-GI foods are thought to increase satiety by prolonging the availability of glucose in the post-absorptive state and by producing a lower insulin response; however, this association remains controversial (14).

In addition, research has also focused on the metabolic responses to dietary proteins. Experimental studies have shown that proteins, such as whey protein, reduce short-term appetite, food intake, and attenuate blood glucose responses (15, 16). It is speculated that dietary proteins enhance satiety and suppress food intake in humans when consumed together with carbohydrates, and that this will reduce the subsequent glycemic response (17). Previous studies indicate that the reduction in blood glucose occurs because of digestion of proteins with a high content of branched-chain amino acids (BCAAs) result in rapid insulin release (15). As a result, research regarding the potential of BCAAs to reduce the postprandial glycemic response and its health benefits has received considerable attention. One promising strategy to minimize postprandial glycemia could be a preload, in which a small load of macro-nutrients, given at a fixed interval before a meal, will induce the Incretin response. The release of peptides, such as GLP-1, GIP, and cholecystokinin (CCK), will slow gastric emptying and stimulate insulin secretion in advance of the main nutrient load at ordinary meals. Studies have shown that fat and protein preloads markedly reduce postprandial glycemic excursions in patients with type 2 diabetes by such mechanisms (18, 19).

However, preloads may potentially increase energy intake, therefore preloads that entail minimal additional energy would be advantageous. Based on the history of preloads used to treat diabetes, we hypothesized that the Inzone Preload (a macronutrient and protein-enriched blend with low-GI) would reduce postprandial glycemia in type 2 diabetic patients. To test this hypothesis, we performed a clinical study, which aimed to explore the potential effects of the Inzone preload on T2DM in Chinese patients. Measurements were performed at both baseline and after 12-week follow-up. The primary purpose of this study was to detect the effect of the Inzone Preload on glucose metabolism. The secondary objective was to assess body weight (BW), lipid profiles, inflammation, insulin resistance, and appetite sensations.

## Materials and Methods

### Study design

The study was designed as a before–after intervention study of 12-week duration with the Inzone Preload given 30 min before each of the three main meals. The study was carried out at the Metabolic Disease Hospital, Tianjin Medical University. Subjects were given written and oral information about the study and gave their written consent to be part of the study. The Ethical Committee of the Tianjin Medical University approved the study as being in accordance with the Helsinki II Declaration. To minimize the potential confounding effects of different anti-diabetic medications, hypoglycemic agent dosages were not changed during the study.

### Study subjects – recruitment and screening

Volunteers were recruited from endocrinology outpatient clinics in Tianjin Medical University Metabolic Hospital. Of total, 19 men and 11 women aged 54.8 ± 7.8 years with a BMI of 26.5 ± 3.4 kg/m^2^ were enrolled in the present study. Participants inclusion criteria were as follows: age >18 years, diagnosis of type 2 diabetes, hemoglobin A1c (HbA1c) ≤9%, postprandial 2 h blood glucose ≥10.0 mmol/L, management of their diabetes with a stable diet, and anti-diabetic medication stable for at least 2 months. Exclusion criteria were as follows: use of pre-meal insulin therapy, any known chronic illnesses (such as liver and kidney disease), allergy to beans or milk. Among the 30 subjects, 19 (63.3%) had abdominal obesity (based on waist circumference), 17 (56.7%) were overweight (BMI >24 kg/m^2^), and 14 (46.7%) had dyslipidemia. Additional characteristics of the study subjects included: tobacco use 7 (23.3%), alcohol consumption 2 (6.7%), and exercise 14 (46.7%).

Subjects were interviewed concerning general health, personal and family health history, eating and exercise habits to assess eligibility, and data on BW, height, waist circumference, blood pressure, and fasting biochemical markers were obtained. Participants were screened using a phone or an in-person meeting. At the initial visit and every 2 weeks for the next 12 weeks, FBG and the postprandial 2 h blood glucose (2 h-BG) were measured. 2 h-BG was measured after instructing the patient to consume a meal containing calories calculated according to their BW. BW and waist circumference were measured in 4 weeks interval. Participants were documented regarding the Inzone Preload tolerance or adverse effects, medications consumed, physical activity, illnesses, and any other information they thought was relevant.

### The inzone preload

The Inzone Preload consists only of natural food ingredients (pea-protein, whey protein, egg albumin, Ω 3/6 fatty acids, whole eggs, apple, rosehip, and sugar beet fiber). Each serving of Inzone Preload (18 g) contains 7.6 g protein, 1.8 g fat (saturated and unsaturated fat are 0.6 and 2 g, respectively), 1.5 g fiber, and 5.2 g carbohydrates, which provided 71 kcal energy (Indevex Biotech, Sweden). Subjects were instructed to mix and shake one package of Inzone Preload powder with 150 mL cold drinking water and after 1–2 min of hydration, to consume the preload 30 min before each meal in the study. The GI value of the preload was <20, as recommended by Brouns et al. in their description of GI methodology.

### Anthropometric measures

Body weight was measured in fasting participants on weeks 0, 4, 8, and 12 using a calibrated digital scale (Tanita TCS-WB-3000, UK) with participants wearing light clothes without shoes. Height was measured without shoes using a stadiometer to the nearest 0.5 cm. Systolic and diastolic blood pressures were measured in duplicate on the left arm after 5 min of rest using an automatic blood pressure monitor (UA-767PC Blood Pressure Monitor, A&D Medical). BMI was calculated as BW (kilogram)/height (square meter). Waist circumference was measured at the midway point between the iliac crest and the lowest rib.

### Biochemical analysis

Blood samples were collected at baseline and at end of study after 10 min of rest in the supine position for each participant. Participants were instructed to avoid all foods, beverages, and strenuous activity for at least 10 h prior to blood sampling. Blood was collected into red-top vacutainers with no anticoagulant and left at room temperature for 30 min prior to centrifugation at 4°C for 15 min at 1500 × *g*. A blood sample from fasting participants was also collected into a tube containing EDTA for analysis of HbA1c. Blood for all other analyses was collected in plain tubes. Serum glucose, lipid profiles, liver and renal biochemistry, and C-reactive protein were determined by using the Hitachi 7070 automatic biochemical analyzer (Hitachi Ltd, Japan). HbA1c was analyzed using a high-performance liquid chromatography, ion-exchange chromatography assay (HLC-723G7, TOSOH, Japan). Insulin was measured using a well-established radioimmunoassay in the endocrinology laboratory of Tianjin Medical University Metabolic Disease Hospital. Insulin resistance was estimated by the Homeostasis Model Assessment Index (HOMA-IR), and was calculated using the following formula: HOMA-IR = [fasting insulin (μU/mL) × fasting plasma glucose (mmol/L)]/22.5; HOMA-β-cell = fasting insulin (μU/mL)/[fasting plasma glucose (mmol/L) − 3.5] (20).

### Satiety

A visual analog scale (VAS), as described by Flint et al. (21), which was 100 mm in length with words anchored at each end that expressed the most-positive and most-negative rating, was used to answer questions regarding subjective feelings of hunger and satiety. Questionnaires were completed at 10 fixed time points, respectively, just before and after the Inzone Preload during the study period. For example, the question was “How hungry do you feel at this moment,” which was anchored at the low end with “very hungry” and with the opposing term “not at all” at the high end. Incremental areas under the percent score time curves (AUCs) from 0 to 2 h were calculated using the linear trapezoidal method.

### Adverse event and safety

All subjects had the possibility to report spontaneous adverse events (AEs) on all visiting days, which took place every second week during the whole study. AEs were documented and graded as mild, moderate, or severe conditions. Aside from AEs, other surrogate laboratory safety endpoints monitored were serum nitrogen and creatinine, hepatic transaminases (alanine aminotransferase and aspartate aminotransferase), and blood routine test.

### Statistical methods

Normally distributed data are expressed as mean ± SD and non-normally distributed data median or as numbers and percentages. Non-normally distributed data were log-transformed for use with parametric statistics. Paired *t*-test was used to compare the differences in clinical characteristics between baseline and after intervention. Unpaired *t*-tests were also used to compare baseline variables between Responders and Non-responders in subjects treated with the Inzone Preload. A multiple linear regression model was prepared to simultaneously evaluate the effects of the factors (age, BMI, duration of diabetes, and baseline HbA1c and HOMA-β) on 2 h-BG changes. The statistical analyses were performed using SPSS windows version 18.0, and *p* value <0.05 was considered to be of statistical significance.

## Results

### Participant characteristics

Thirty participants were enrolled in the study between February and September 2013 and a total of 27 completed the study. The reasons for withdrawals were: one disliked the study treatment powders and two went abroad for personal reasons. Of the 27 completing participants, 17 were males and 10 were females. Participants had a mean of 6.3 ± 3.6 years since diagnosis of type 2 diabetes. The average age of participants was 54.8 ± 7.8 years, with an average BMI of 26.5 ± 3.4 kg/m^2^. Baseline characteristics of the study participants are shown in Supplement S1 in Supplementary Material. At baseline, 83.3% (25/30) of the patients were taking two or more medications, the most widely used combination was metformin and repaglinide, followed by metformin and gliclazide. Three percent (4/30) of the patients were using basal insulin.

### Changes in parameters relating to glucose metabolism following 12-week inzone preload intervention

After 12 weeks of the Inzone preload intervention, 2 h-BG was reduced from 12.2 ± 1.2 mmol/L at baseline to 10.5 ± 2.0 mmol/L, mean change of 1.7 mmol/L (95% CI of 0.6–2.4, *p* = 0.013), but no significant changes were observed in FPG; Reductions in 2 h-BG were found near maximal at week 4, with modest progressive reductions and no apparent plateau observed through the 12-week period. Eighteen (67%) of the patients achieved the target 2 h-BG reduction of 1.9 mmol/L at the end of the study. Overall HbA1c values were reduced from 7.4 ± 0.2% at baseline to 7.1 ± 0.2% at 12 weeks, with an average relative change of 5.4% (95% CI 1.0–6.2, *p* < 0.05). Neither the HOMA-IR nor HOMA-β was significantly changed following the 12-week Inzone preload (HOMA-IR from baseline 4.3 ± 1.5 to 4.2 ± 2.1, *p* = 0.670; HOMA-β from baseline 56.4 ± 20.2 to 60.3 ± 24.0, *p* = 0.824) in all the subjects (Table 1).

**Table 1:** **Characteristics of patients (mean ± SD) at baseline and after 12 weeks of preload testing (*n* = 27)**.

Characteristics	Baseline	After 12 weeks	Mean changes from baseline (95% CI)	*p* Value
FBG (mmol/L)	7.2 ± 0.8	7.0 ± 0.6	−0.2 (−0.4 to 0.2)	0.714
2 h-BG (mmol/L)	12.2 ± 1.2	10.5 ± 2.0	−1.7 (−0.6 to 2.5)	0.013
FPI (mIU/L)	18.8 ± 13.5	16.9 ± 10.3	−1.9 (−3.2 to 0.4)	0.390
HbA1c (%)	7.4 ± 0.3	7.1 ± 0.2	−0.3 (−0.5 to −0.1)	0.032
Weight (kg)	76.4 ± 11.5	75.5 ± 10.6	−0.9 (−1.9 to 0.7)	0.604
BMI (kg/m^2^)	26.5 ± 3.4	26.4 ± 3.5	−0.1 (−0.1 to 0.1)	0.672
WC (cm)	94.2 ± 10.5	93.4 ± 10.1	−0.8 (−1.4 to 0.5)	0.529
TG (mmol/L)	1.7 ± 0.6	1.8 ± 0.8	0.1 (−0.3 to 0.2)	0.644
TC (mmol/L)	4.8 ± 0.9	4.3 ± 0.8	−0.5 (−0.2 to −0.7)	0.022
HDL-c (mmol/L)	1.2 ± 0.2	1.2 ± 0.3	0.0 (−0.1 to 0.2)	0.517
LDL-c (mmol/L)	2.8 ± 0.6	2.5 ± 0.4	−0.3 (−0.1 to −0.5)	0.018
CRP (mg/L)	1.5 ± 1.4	0.7 ± 0.7	−0.7 (−0.2 to −1.1)	0.047
HOMA-IR	4.3 ± 1.5	4.2 ± 2.1	−0.1 (−0.2 to 0.3)	0.670
HOMA-β	56.4 ± 20.2	60.3 ± 24.0	2.9 (1.1–11.3)	0.824

*Normally distributed data expressed as mean ± SD and non-normally distributed data were log-transformed for use with parametric statistics. FBG, fasting blood glucose; 2 h-BG, postprandial 2 h blood glucose; FPI, fasting insulin; TG, triglyceride; TC, total cholesterol; LDL-C, low density lipoprotein-cholesterol; HDL-c, high density lipoprotein-cholesterol; CRP, c-reactive protein; HOMA-IR = fasting glucose (mg/dL) × fasting insulin (U/mL)/405; 95% CI, lower 95% confidence interval limit, upper 95% confidence interval limit. HOMA-β = 360 × FPI (μIU/mL)/[FPG (mg/dL) − 63]*.

### Changes in parameters relating to serum lipids and CRP following 12-week inzone preload intervention

Compared with baseline values, overall serum total cholesterol and LDL concentrations were significantly reduced by 0.5 ± 0.2 and 0.3 ± 0.1 mmol/L, respectively. Changes in mean serum triglycerides from 1.7 ± 0.6 to 1.8 ± 0.8 mg/dL did not reach significant levels (*p* = 0.644). In addition, no significant changes were observed in HDL-c during the intervention period (*p* = 0.724). Interestingly, the levels of serum CRP, a marker of clinical inflammation, were significantly reduced from baseline following 12 weeks Inzone Preload intervention, with mean changes from 1.5 ± 1.4 to 0.7 ± 0.7 mg/L (*p* = 0.047*)* (Table 1).

### Changes in anthropometric measures following 12-week inzone preload intervention

At the end of the 12-week intervention period, BW was reduced from 76.4 ± 11.5 kg at baseline to 75.5 ± 10.6 kg, mean change of 0.9 kg (95% CI of −1.9 to 0.7, *p* = 0.390) (Table 1). Thirteen patients (48.1%) lost more than 3% BW during the intervention phase, from 83.3 ± 12.0 to 80.8 ± 10.7 kg. Three subjects gained weight during the intervention period (79.3 ± 14.4 vs. 80.0 ± 9.4 kg, *p* = 0.613) and the remaining 11 subjects did not show any change in weight. Most of the weight loss was observed during the initial month, with progressive reductions through week 12 (Supplement S2 in Supplementary Material). BW reduction was linked to changes in waist circumference (*r* = 0.524, *p* = 0.035). Compared with women, men had a higher mean baseline BW; however, we found no significant sex differences for changes in weight (data not shown).

### Changes in VAS ratings of satiety and hunger

**Figure 1:**
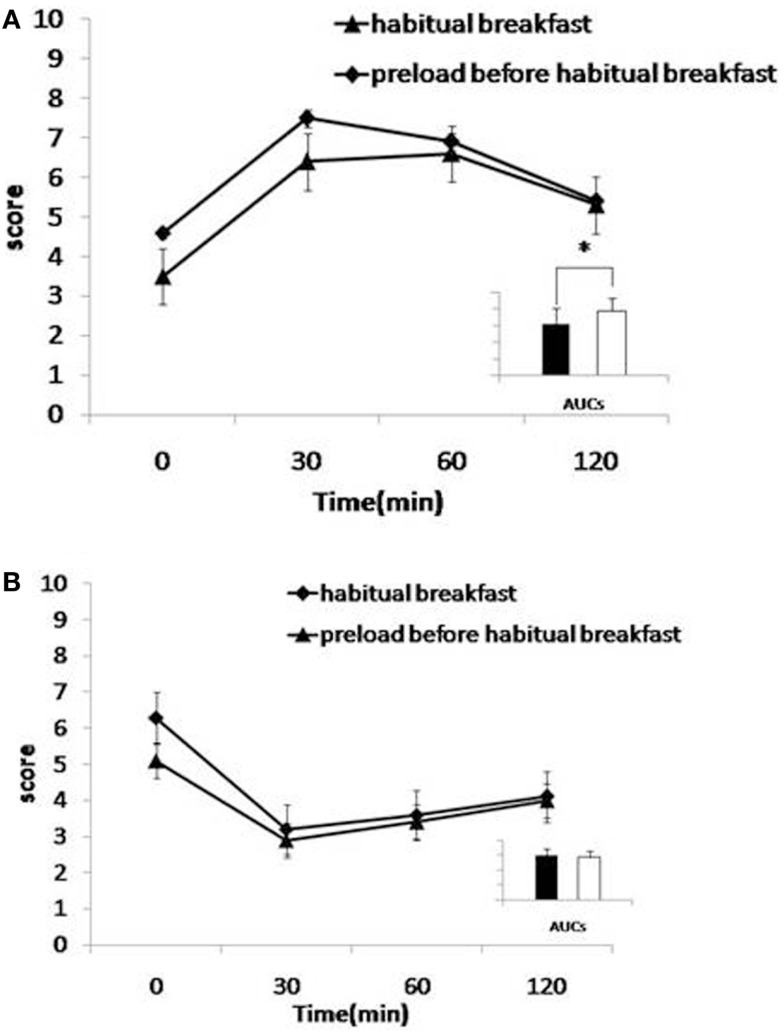
**(A,B)** Effects of preload on satiety **(A)** and hunger **(B)**. Mean visual analog scale (VAS) subjective scores for satiety **(A)** and hunger **(B)** for habitual breakfast. Habitual breakfast is indicated by -◆- and Preload before habitual breakfast by -▲-. Inserts: area under the curve histograms for 0–120 min are shown, habitual breakfast (open bars), and preload before habitual breakfast (filled bars). The time-by-interaction was statistically significant for satiety (*p* < 0.05, 0–120 min).

The Inzone Preload intervention resulted in a significantly higher postprandial satiety at the onset of the meal (at time point 0 min, 30 min after preload was given) (*p* = 0.045) and 30 min (*p* = 0.042) after the habitual breakfast (Figure 1A). The AUCs for satiety were not significantly larger at any time after Inzone Preload than habitual breakfast. Regarding the questions on aspects of hunger, subjects reported a lower level of hunger after the Inzone Preload than after the habitual breakfast at 0 min (*p* = 0.047) and after 30 min (*p* = 0.352). No differences were observed in the AUCs for hunger (*p* = 0.241) (Figure 1B).

### Side effects and safety

There were few AEs during the study and these were generally mild. One subject reported mild diarrhea on the initial, but this phenomenon disappeared after a week, the subject remained compliant throughout the study. There were no significant changes in the blood routine examination, serum nitrogen and creatinine, hepatic transaminases compared to those at the baseline (data not shown).

### Identification of subgroups and multiple linear regression analysis

**Figure 2:**
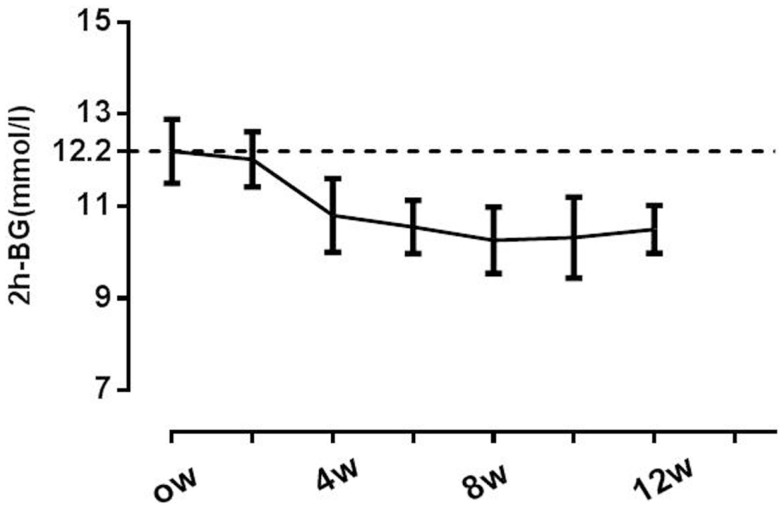
**Changes in postprandial glucose throughout the study**. A lowering of 2 h-BG of ≥1.9 mmol/L was used as a cut-off separating 67% of the subjects as responders. Responders (*n* = 19) are indicated with -◆-. Non-responders (*n* = 8) are indicated with -▲-.

It was clear from the data above that the response to Inzone treatment varied between individuals. In an attempt to analyze data further, we attempted to separate individuals responding from non-responders using 2 h-BG measurements. A lowering of 2 h-BG ≥1.9 mmol/L (22) was used as a cut-off and this separated 67% of the subjects into one group termed responders (*n* = 19) and another group called non-responders (*n* = 8) (Table 2). In the responding group, reduction of 2 h-BG was near maximal at week 4, with modest progressive reductions and no apparent plateau observed through week 12 (Figure 2). In the responding group, a reduced BW was seen in 13 patients after 8 weeks and this was maintained during the study (Supplement S2 in Supplementary Material). A multiple regression analysis was performed to evaluate the independent factors that might predict 2 h-BG changes. A weak but significant linear correlation was found that shorter duration of diabetes, higher HOMA-β level and greater reduction in BW were significantly correlated with 2 h-BG reduction, but BMI, baseline HbA1c were insignificant factors (Table 3).

**Table 2:** **Baseline and change of biochemical and clinical variables during the study period (end of the study minus baseline) for the Responders group and Non-responders group**.

variables	Responders (*n* = 19)		Non-responders (*n* = 8)		*p* Value
	Baseline	Change	Baseline	Change	
Age	54.0 ±7.8	–	56.0 ± 6.5	–	0.064
Duration of diabetes (years)	4. 3 ± 3.6	–	7.6 ± 2.7	–	0.042
Weight (kg)	73.4 ± 11.5	−1.6 ± 0.3	76.5 ± 10.6	−0.1 ± 0.2	0.011
BMI (kg/m^2^)	26.2 ± 6.4	0.1 ± 0.1	26.9 ± 3.1	0.0 ± 0.1	0.587
FBG (mmol/L)	7.0 ± 1.1	−0.3 ± 0.2	7.4 ± 1.0	−0.2 ± 0.1	0.882
2 h-BG (mmol/L)	12.0 ± 1.2	−2.2 ± 0.9	12.5 ± 1.8	−0.7 ± 0.3	0.007
HbA1c (%)	7.3 ± 0.8	−0.4 ± 0.2	7.7 ± 1.1	−0.1 ± 0.1	0.048
TG (mmol/L)	1.5 ± 0.6	0.1 ± 0.2	1.9 ± 0.6	0.1 ± 0.2	0.330
TC (mmol/L)	4.6 ± 0.9	−0.6 ± 0.2	5.0 ± 0.8	−0.5 ± 0.4	0.587
HDL-c (mmol/L)	1.2 ± 0.3	0.1 ± 0.1	1.0 ± 0.3	0.0 ± 0.1	0.724
LDL-c (mmol/L)	2.7 ± 0.7	−0.3 ± 0.3	3.0 ± 0.8	−0.3 ± 0.2	0.936
CRP (mg/L)	1.4 ± 1.4	−1.1 ± 0.4	1.8 ± 1.6	−0.2 ± 0.3	0.021
HOMA-IR	4.1 ± 2.5	−0.6 ± 0.2	4.9 ± 3.1	−0.2 ± 0.4	0.342
HOMA-β	56.4 ± 20.2	10.5 ± 3.6	30.3 ± 24.0	6.4 ± 4.7	0.572

*Normally distributed data expressed as mean ± SD and non-normally distributed data were log-transformed for use with parametric statistics*.

**Table 3:** **Multiple regression analysis of the potential variables for predicting changes in 2 h-BG level as dependent variables**.

Variable	β	*p* Value
BMI (kg/m^2^)	0.046	0.263
Duration of diabetes	−0.511	0.031
Baseline HbA1c (%)	0.499	0.451
Δ Weight (kg)	0.964	0.044
HOMA-β	0.240	0.039
HOMA-IR	0.610	0.347

*Beta is the standardized partial regression coefficient of multiple linear regression analysis*.

## Discussion

To our knowledge, this study is first to investigate the long-term metabolic effects of a macro-nutrient preload in T2DM. No adverse effects were noted among the 27 subjects who completed the study. As a dietary intervention, a majority of Inzone Preload-treated T2DM patients responded by lowing 2 h-BG, HbA1c, total cholesterol and LDL, and CRP at the end of 12-week treatment period. It was also noteworthy that significant weight loss occurred in 13 of the 27 subjects (48.1%) in the study. Other studies have evaluated the metabolic effects of different types of preloads, and a common feature of those studies is the testing of simple ingredients and short observation times. In contrast, our study concerns a macro-nutrient preload mix and effects were evaluated during a relatively long time. The components of the Inzone Preload are a mix of natural food ingredients, and in addition to high protein and low carbohydrate, the preload also contains polyunsaturated fatty acids, dietary fiber, and vitamin C, which have documented health benefits (23–26).

Our study showed that mean 2 h-BG and HbA1c levels are significantly decreased by preload treatment amounting to 1.7 mmol/L and 0.3%, respectively. This was observed in a majority of subjects whereas some patients responded poorly to the treatment. The reason why not all patients responded is not clear but there were differences in T2D characteristics within the group and the compliancy to treatment was not monitored. The present study supports previous findings that protein-enriched and low-GI diets can reduce 2 h-BG and HbA1c (16, 17). However, this effect on 2 h-BG lowering was not observed in all subjects in our study where 19 subjects (70.4%) displayed a decrease in 2 h-BG. We stratified the subjects into two subgroups to analyze this phenomenon. Compared to the non-responding subgroup at baseline, the responding subgroup had a shorter duration of diabetes, higher HOMA-β level, indicating that Inzone preload may be more beneficial in less advanced diabetes. A decrease in postprandial glucose by a macro-nutrient preload might be explained by the Incretin response where increased GLP-1 can affect plasma insulin and insulin sensitivity (27). Our data also suggest that preload induced changes in 2 h-BG levels positively correlate with weight loss. Weight loss can induce 2 h-BG reductions through improving insulin resistance (28). The perceived satiety increase observed in this study can be another reason for the postprandial glycemia reduction, since postprandial glycemia is directly associated with appetite (29, 30).

High-protein and low-GI diets can be effective in weight control of obese subjects (31) as well as in T2DM patients (31–33). In this study, BW and waist circumference tended to decrease at the end of the 12-week period, although not reaching statistical significance. However, 13 (48.1%) lost weight with a mean change of −1.7 kg (3% weight loss) and maintained progressive reduction during the intervention phase. The relatively small sample size together with short intervention period might have limited our ability to detect significant weight loss. Weight reduction in T2DM patients can be a challenge (34), but even a modest weight reduction of <5% is of clinical importance, since glycemic metabolism and cardiovascular disease risk factors improved with net weight loss of as little as 2–5% in persons with T2DM (35). Possible explanations for weight loss related to intake of proteins include a rise in thermic effects (32, 36, 37) and energy intake reduction due to the increased satiety (31, 38), as satiety is an important factor in the regulation of food intake and in the control of weight (28).

Low-GI or protein diets can have positive effect on blood lipids (39–42), but the combination of low-GI and high-protein diets on blood lipids are somewhat controversial. In a 28-day high-protein low-GI diet intervention program, total cholesterol and LDL cholesterol were significantly reduced (43). But in another study, these lipid parameters did not change after 4 weeks of high-protein low-GI diets intervention (27). Our results suggest that Inzone Preload reduces total cholesterol and LDL cholesterol. Another important finding in our study is that the Inzone Preload significantly reduced the inflammatory marker CRP, which is relevant in consideration to the connections between inflammation and T2D. The results reported here are consistent with other published interventions demonstrating that low-glycemic and high-protein diets reduce serum CRP (44). The CRP reduction may not only be due to glucose improvement (45) but may also involve polyunsaturated fatty acids and dietary fiber in the preload, since most of these components are known to reduce CRP (46–48). It should be emphasized that the present study concerns the concept of preload, which is different from complete dietary change. There are certain advantages with the preload concept because the challenges to completely change diet are not needed and also that preload effects can be studied much in the same way as regular drug testing.

Our study had several limitations. First, the study was not a randomized clinical trial. Second, the study was performed in outpatient clinic, so we could not monitor diets and other life style factors of our subjects throughout the study period, which might have impacted study outcomes. Finally, the relatively small sample size and short duration of the study limited our power to detect differences in many metabolic parameters.

In summary, the results of this study support our hypothesis that Inzone preload improves glycemic control and have favorable effects on weight and serum lipids in patients with T2DM. Despite some potential limitations, our preliminary study offers new insights into patients with sustained poor 2 h-BG control who might benefit from this type of interventions. As a dietary strategy, the Inzone preload, consisting of polyunsaturated fatty acids, dietary fiber from natural food ingredients has unique advantages in the lipid and inflammation improvement. We recommend large studies over a longer period of time to investigate the longitudinal effects of the Inzone preload on diabetic patients.

## Conflict of Interest Statement

The authors Norstedt and Sederholm are also consultants for Indevex AB in Sweden. The remaining co-authors declare that the research was conducted in the absence of any commercial or financial relationships that could be construed as a potential conflict of interest.

## Supplementary Material

The Supplementary Material for this article can be found online at http://journal.frontiersin.org/article/10.3389/fendo.2015.00139
