# Supplementary material for: Effects of a Macro-Nutrient Preload on Type 2 Diabetic Patients
Source: Front Endocrinol (Lausanne). 2015 Sep 16;6:139. doi: 10.3389/fendo.2015.00139 (PMC4584965; doi:10.3389/fendo.2015.00139)
Supplement: Supplementary file 1 [file Data_Sheet_1.DOCX]

**Supplement S1. Table 1. Baseline characteristics of participants**

| Characteristics | Baseline(n=30) |
| --- | --- |
| sex(Men/Women) | 19/11 |
| age, years | 54.8 ± 7.8 |
| weight, kg | 75.0 ± 12.1 |
| BMI, kg/m^2^ | 26.5 ± 3.4 |
| WC,cm | 93.0 ± 10.5 |
| duration of diabetes, years | 6.3 ± 3.6 |
| SBP,mmHg | 137.2 ± 14.3 |
| DBP,mmHg | 86.4 ± 15.3 |
| FPG, mmol/l | 7.2 ± 0.8 |
| FPI, pmol/l | 19.0 ± 17.1 |
| HbA1c, % | 7.4 ± 0.2 |
| Medication before admission |  |
| Met | 24/30( 80.0%) |
| SU | 22/30 (73.3%) |
| α-GI | 18/30 (60.0%) |
| TZD | 5/30 (16.7%) |
| Basal insulin | 4/30 ( 13.3%) |

**Supplement S2. Fig S2 Body weight during the study**


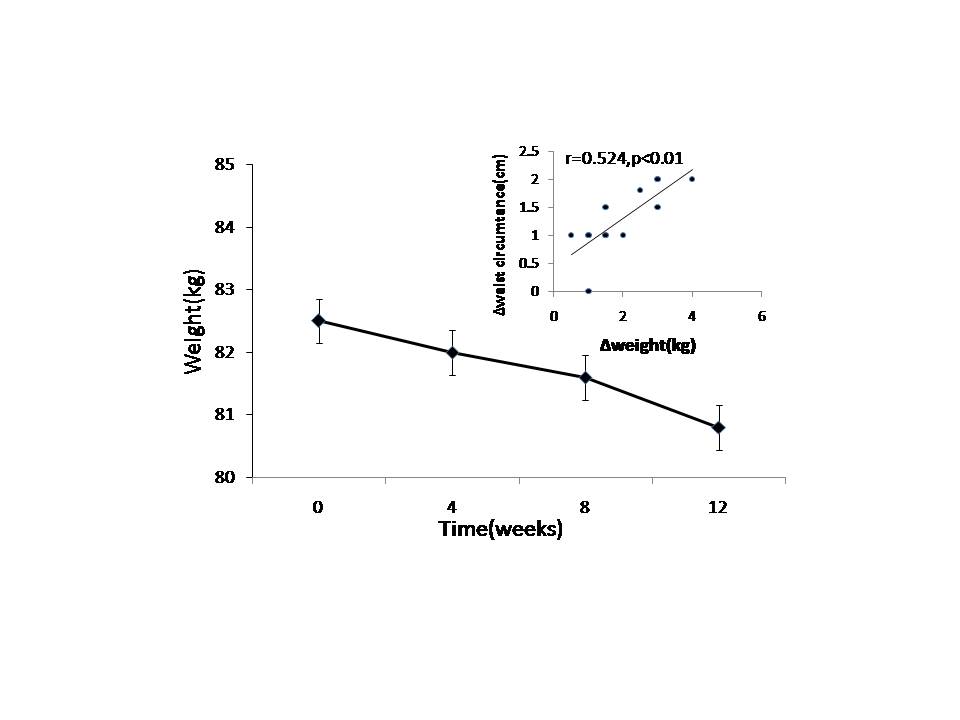


S2 Body weight was recorded each month. The figure depicts the 13 subjects that reduced more that 3% body weight (13 out of 27 subjects). Insert: Simple univariate analysis was used to test the significance of the association between weight and waist circumference.
